# Histopathological Analysis from Gallic Acid Administration on Hippocampal Cell Density, Depression, and Anxiety Related Behaviors in A Trimethyltin Intoxication Model 

**DOI:** 10.22074/cellj.2016.3838

**Published:** 2016-01-17

**Authors:** Marzieh Moghadas, Mohammad Amin Edalatmanesh, Reza Robati

**Keywords:** Trimethyltin, Gallic Acid, Hippocampus, Cell Density

## Abstract

**Objective:**

The present study investigated the effects of gallic acid (GA) administration on trimethyltin chloride (TMT) induced anxiety, depression, and hippocampal neurodegen- eration in rats.

**Materials and Methods:**

In this experimental study, the rats received intraperitoneal (i.p.) injections of TMT (8 mg/kg). The animals received either GA (50, 100 and 150 mg/kg) or saline as the vehicle for 14 consecutive days. We measured depression and anxiety levels of the rats by conducting the behavioral tail suspension (TST), elevatedplusmaze (EPM), and novelty suppressed feeding (NSF) tests. Histological analyses were then used to de- termine the cell densities of different hippocampal subdivisions. The data were analyzed with ANOVA and Tukey’s post hoc test.

**Results:**

GA administration ameliorated anxiety and depression in the behavioral tests. The cell densities in the CA1, CA2, CA3 and DG hippocampal subdivisionsfrom GA-treat- ed rats were higher than saline treated rats.

**Conclusion:**

GA treatment against TMT-induced hippocampal degeneration altered cellular loss in the hippocampus and ameliorated the depression-anxiety state in rats.

## Introduction

Trimethyltin chloride (TMT, C3H9ClSn) is found mainly in polyvinyl chloride (PVC) and silicone products such as kitchen utensils, food packaging, and pesticides (1). This organotin compound can be neurotoxic and produces neuronal degeneration in both human and rodent central nervous systems (2). TMT toxicity causes behavioral and cognitive disorders in humans and laboratory animals (3,4). As TMT-induced neurodegenerative phenomena have crucial pathogenic pathways in common with a majority of neurodegenerative disorders, including selective neuronal death and neuro-inflammation, it is seen as an effective way to obtain a neurodegenerative animal model. The neurotoxic effects of this compound include limbic-cerebellum syndrome in humans which is related to amnesia, confusion, epilepsy, buzzing ears, insomnia, and depression (5). Specialized neuronpopulations’ selective vulnerability to the effects of TMT neurotoxicityon the limbic nervous system, in particular the hippocampus, can be employed as an effective model to explore behavioral changes that result from neurodegeneration. These changes include attention deficits hyperactivity disorder (ADHD), aggression, cognitive impairment, and depression-like behavior (6). 

There has been tremendous effort to develop beneficial agents from medicinal plants with the intent to achieve an optimal level of neuroprotection. Attention has been paid to a wide variety of natural antioxidants that can scavenge free radicals and protect cells from oxidative damages, such as resveratrol and catechins (7,8). Gallic acid (GA) or 3,4,5-trihydroxybenzoic acid, one of the most important polyphenolic compounds, can be foundin plants. It serves as a putative active compound in tannin. GA and its derivatives are considered to be polyphenyl natural products particularly found in processed beverages such as red wine and green tea (9). GA induces anti-oxidant, anti-inflammatory, anti-microbial, and anti-cancer activities (10,12). GA-containing plant extracts have been reported to contain anti-diabetic, anti-angiogenic and anti-melanogenic effects in addition to reducing the incidence of myocardial infarction, and oxidative liver and kidney damageas (13,15). GA also protects neural cells against *in vitro* b-amyloid peptide (Aβ)-induced death (16). It can be used further as an antioxidant in foods, cosmetics, and in pharmaceuticals (17). GA is nontoxic to mammals at pharmacological doses. The lethal dose at which 50% of the animals died (LD50) for GA is 5 g/kg body weight in rats (18). The present study aims to explore the neuroprotective effects of GA on TMT intoxication and hippocampal neurodegeneration in rats. 

## Materials and Methods

### Experimental procedures

For this experimental study, we used adult male Sprague Dawley rats (220 ± 20 g). Animal handling and all related procedures were carried out in accordance with the Animal Ethical Committee Acts of Islamic Azad University, Shiraz Branch. All animals were randomly housed with five rats per cage at a temperature of 21˚C and 65% humidity with a 12-hour light/dark cycle (lights on at 07:00 am). Water and food were freely available throughout the study. 

### Animals

The rats were divided into 5 groups of 10 rats per group, as follows: an intact control group that did not receive any treatment; vehicle treated group which received saline after TMT intoxication (TMT+saline); and three experimental groups which received either 50, 100, or 150 mg/ kg body weight GA (TMT+GA50, TMT+GA100, TMT+GA150) after intoxication. 

### Trimethyltin intoxication and gallic acid treatment

A single dose (8.0 mg/kg body weight) of TMT (Sigma, Germany) dissolved in 0.9% saline (vehicle) was injected intraperitoneally (i.p.) into the rats. The animals were returned to their cages. One week after TMT intoxication, the animals received different doses of GA (Sigma, Germany) that were administered i.p. on a daily basis for two weeks. 

### Behavioral observations

#### Tail suspension test (TST)

Each animal was suspended by its tail from a horizontal bar (located 50 cm from the floor) by scotch tape in a 25×30×5 cm box. Animals were suspended for six minutes in order to record the duration of behavioral parameters such as immobility time. The rats showed several escape-oriented behaviors along with temporary increasing bouts of immobility. 

#### Elevated plus maze (EPM)

Anxiety-related behavior was evaluated using the standard EPM test. The maze consisted of two opposing open arms (50×10 cm) and two opposing arms enclosed with 40-cm high walls, elevated 50 cm above the floor. Each rat was placed for 5 minutes in the apparatus for acclimatization prior to conducting the tests. The tests facilitated exploratory behaviors. The rats were observed by an examiner who sat about 2 m from the apparatus. The exposed rats were placed in the center of the EPM facing one of the open arms. Rats were placed immediately after the pretest. Lower levels of anxiety were indicated by measures of anxiety-related behavior such as the total time spent in the open and closed arms, with a longer time in the open arms. The researchers also checked locomotor activity levels by measuring the total traveled distance. The maze was cleaned after each trial (19). 

#### Novelty suppressed feeding (NSF)

The groups were deprived of food for 24 hours before being placed in the corner of the open field arena. Accordingly, they were left to explore for 10 minutes. Next, a food-containing pellet was put in the center of the new environment. The latency of each rat when leaving the corner to reach the food was recorded. After the recording process, the rats were put back in their home cages and allowed to eat pre-weighed food. The food intake was recorded after 5, 15, and 30 minutes as a measure of appetite drive (20). 

### Histological studies

All rats were totally anesthetized using a mix of 1% ketamine (30 mg/kg, Alfasan, Netherland) and xylazine hydrochloride (4 mg/kg, Alfasan, Netherland) immediately following treatments and behavioral assessments. Then, 100 ml of saline followed by a 100 ml fixative solution [3% glutaraldehyde (Merck, Germany)+10% paraformaldehyde (Merck, Germany) in 0.2 mol buffer phosphate (Sigma, Germany) at pH=7.4 and 40˚C] was administered to the animals for a one-hourperiod. The animals’ brains were carefully removed, histologically processed, and fixed. Finally, the specimens were dehydrated in an ascending ethanol series, cleared with xylene, and placed in paraffin. The paraffin blocks were cut into 5 µm-thick serial coronal sections. 

Next, we chose ten uniform random sample sections that included hippocampus from each brain. The sections were mounted on slides for hematoxylinand eosin (H&E) staining and quantification of cell density in the hippocampal subdivisions (6). 

### Quantification of degenerated neurons and cell density

We calculated the number of cell density per unit area (NA) of the CA1, CA2, CA3, and DG hippocampal subdivisions (n=5 for all groups). The sections were studied under a light microscope by a ×40 objective lens (UPlanFI, Japan). The obtained images were then moved to a computer using a high-resolution camera (BX51, Japan). 

Digital photos of all sections were taken and we calculated the numbers of neurons in a 10000 µm^2^counting frame, then calculated the mean scores of neurons NA in different regions of the hippocampus according to the following formula: 

NA=(ΣQ)(a(fΣp)

Where, ΣQ is the sum of counted particles present in the sections, a/f is the area associated with each frame, and ΣP is the sum of frame associated points hitting space (21). 

### Statistical analysis

The data are expressed as mean ± SEM. Statistical differences between group means were evaluated by one-way ANOVA followed by Tukey’s post hoc test. P<0.05 was considered statistically significant. 

### Results

#### Depression-like behaviors in the tail suspension test

**Fig.1:**
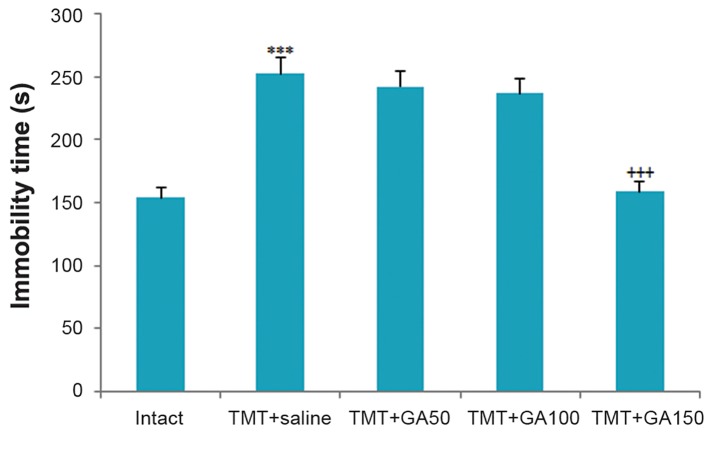
Immobility time during TST. The TMT+saline group displayed a significant difference compared to the intact group (***; P<0.001). Significant differences were observed in the TMT+GA150 group compared with the TMT+saline group (+++; P<0.001). All error bars indicate the SEM. TST; Tail suspension test, TMT; Trimethyltin chloride and GA; Gallic acid.

The results of the ANOVA revealed significant differences among the TMT+saline and intact rats during the period of immobility in the TST. The TMT+saline group had significantly higher immobility compared to the intact controls as shown in figure 1 (F _4,45_:57.512,P<0.001). In addition, there was a significant difference among the GA-treated groups that received 150 mg/kg body weight GA compared with the control group in terms of mean animal immobility (Fig .1, F_4,45_:93.158, P<0.001).

### Decreased anxiety-related behavior in the elevated plus maze

**Fig.2:**
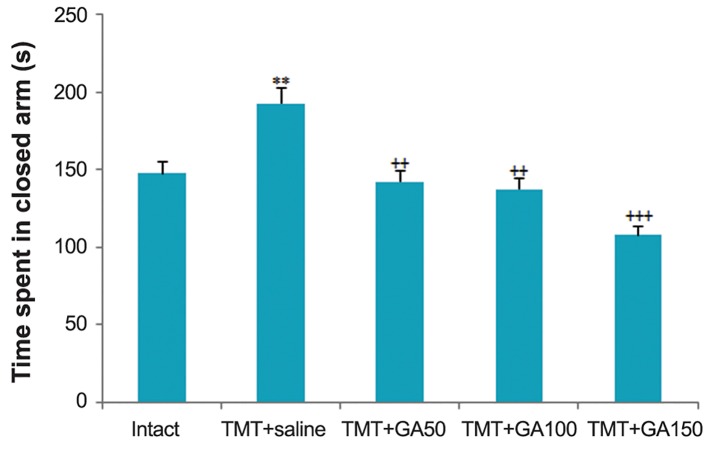
Time spent in closed arm during EPM. GA administration displays as an anxiolytic factor for post-TMT intoxication. The results revealed significant differences among the TMT+saline and intact groups (**; P<0.01). GA-treated rats also spent less time in the closed arms compared to the TMT+saline group from the third week after intoxication. All error bars represent the SEM (++; P<0.01 and +++; P<0.001). EPM; Elevated plus maze, GA; Gallic acid and TMT; Trimethyltin chloride.

We examined anxiety levels in the EPM. The results of one-way ANOVA of the total time spent in the closed arms of the EPM indicated significant differences between TMT+saline and the intact group (Fig .2,F_4,45_:23.808,P<0.01).

The rats that received different doses of GA spent a shorter time in the closed arms compared to the TMT+saline group for 3 weeks after intoxication. This finding accounted for the reduced anxiety in the TMT+GA50, TMT+GA100, and TMT+GA150 groups (Fig .2,F_4,45_:45.98,P<0.01). The differences among GA-treated rats and non-operated controls did not result from differences in anxiety levels as both groups experienced an equal amount of total time spent in the closed arms (Fig .2). 

### Anxiety-like phenotype in the novelty suppressed feeding test

**Fig.3:**
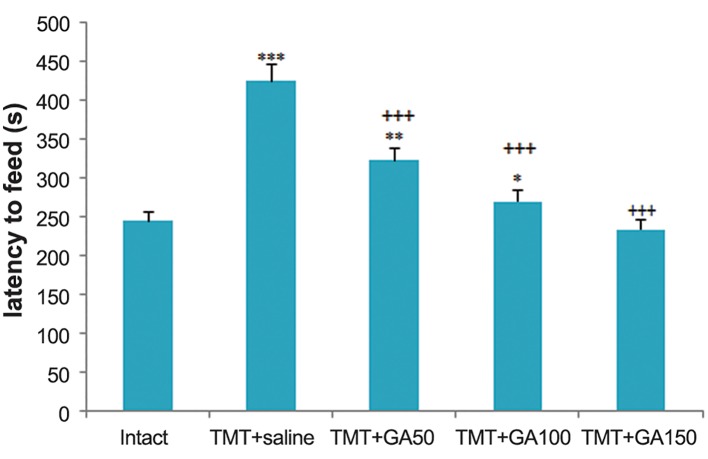
Delay time to eat food during the NSF test. There were significant differences between the TMT+saline and intact groups (***; P≤0.001). As the results of the NSF test suggested, GA administration took shorter to feed in the new environment compared to the TMT+saline group (+++; P<0.001). There were significant differences between the TMT+GA50 (**; P≤0.01) and the TMT+GA100 (*; P≤0.05) compared to the intact control rats. Data are presented as mean ± SEM. NSF; Novelty suppressed feeding, GA; Gallic acid and TMT; Trimethyltin chloride.

Based on the results of the behavioral analyses, we observed that TMT intoxication induced a significant anxious-like phenotype according to the tests that specifically assessed anxiety-like behavior. This anxious phenotype was then confirmed in contextual behavioral paradigms that included the NSF test. The TMT+saline group had a greater delay in starting to eat food compared to the intact controls (Fig .3, F_4,45_: 78.943, P<0.001)On the other hand, the GA-treated rats displayed a shorter latency time to eat in the open field arena compared to the TMT+saline group (Fig .3, F_4,45_:34.371, P<0.001).

### Histology

**Fig.4:**
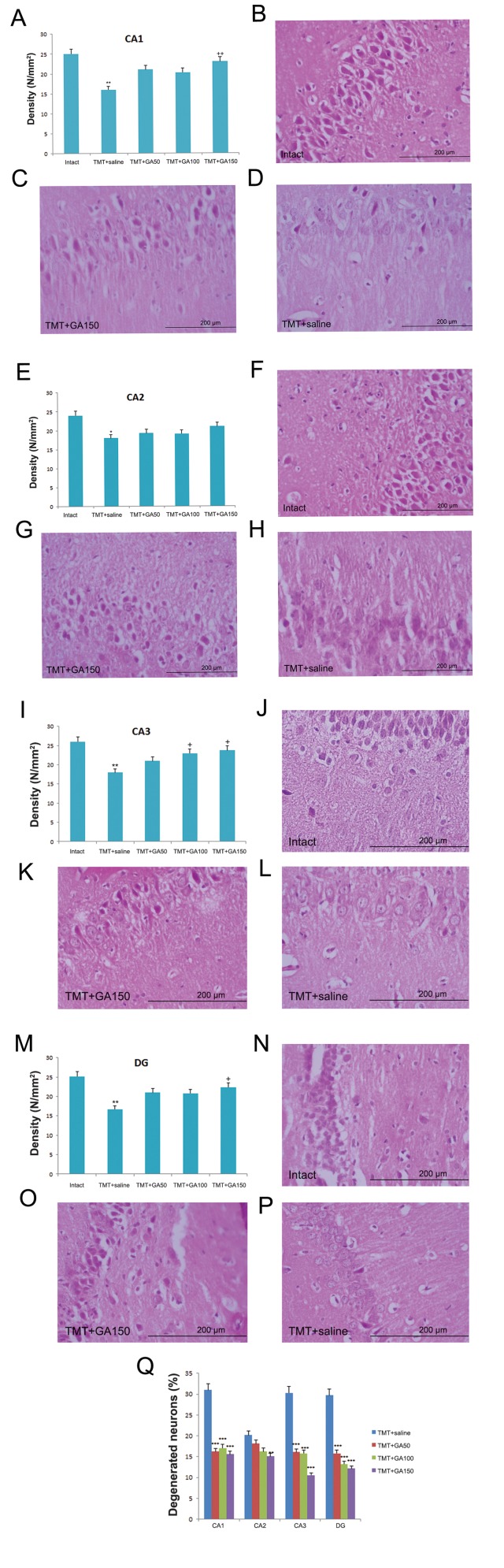
A-P. A comparison of cell density and percentage of degenerated cells in the hippocampal subdivisions. In TMT treated rats, there was decreased cell density in the CA1, CA3, DG (**; P≤0.01) and CA2 (*; P≤0.05) hippocampal compared to the intact controls. However, GA administration ameliorated this finding in the CA1, CA3 and DG hippocampal subdivisions (++; P≤0.01 and +; P≤0.05) compared to the TMT+saline group and Q. The percentage of degenerated neurons decreased in GA treated groups (***; P≤0.001) compared to the saline treated group. Photomicrograph shows cell density in B. CA1, F. CA2, J. CA3, and N. DG areas of the rat hippocampus in the intact control, TMT+saline [D. CA1, H. CA2, L. CA3 and P. DG] and TMT+GA150 [C. CA1, G. CA2, K. CA3 and O. DG] groups. H&E staining. TMT; Trimethyltin chloride, GA; Gallic acid and H&E; Hematoxylin and eosin.

Although we observed karyorrhexis, cell membrane destruction, and apoptosis in both the saline and GA treated groups, these lesions were more frequent in the TMT+saline group compared to the TMT+GA150 group (Fig .4). Comparisons between the intact control and vehicle treated groups revealed that TMT caused reduced cell density in the CA1, CA2, CA3 and DG hippocampal subfields (Fig .4,F_4,45_: 24.67, P<0.05).The cell density in the CA1 (TMT+GA150), CA3 (TMT+GA100 and TMT+GA150), and DG (TMT+GA150) subdivisions of the hippocampus increased compared to the TMT+saline group (Fig .4,F_4,45_: 19.64, P<0.05).The percentage of degenerated cells used as an estimate of post-TMT intoxication damage was significantly lower in the TMT+GA50, TMT+GA100, and TMT+GA150 groups compared to the saline treated group in all hippocampal subdivisions (Fig .4Q,F_4,45_ :28.13,P<0.001).

## Discussion

Previously, it has been reported that TMT administration leads to depression-like behaviors (6). TMT-toxicity also causes disorders in learning and memory due to neuroatrophy in the limbic system, especially in the hippocampus (22,23). Due to the common pathogenesis of these disorders and their relationships with mood disorders, there is a possible chance of depressive-anxious like behaviors (6). Studies on neurodegeneration caused by TMT have shown that TMT toxicity could decrease neurotophines and induce apoptosis in different areas of the central neurological system such as the hippocampal regions (6,24). Jenkins and Barone have provided evidence that shows the importance of a signaling cascade involving caspase activation, protein kinase p38, and oxidative stress in apoptosis induced by TMT (25). 

GA, as a natural product of tannin hydrolysis, is found in large quantities in grapes, tea, various berries, and other fruits, as well as in wine (26). GA has been found to exert neuroprotective effects on amyloid β-mediated neurotoxicity (16), lead nitrate, streptozotocin, kainic acid, and/or 6-hydroxy dopamine (6-OHDA)-induced-brain oxidative damages (27,30). Given its ability to scavenge ROS such as superoxide anions, hydrogen peroxide, hydroxyl radicals, and hypochlorous acid, GA has received much attention. GA anti-mutagenic and anti-carcinogenic activities have also added to its popularity (31,33). In addition, GA has been shown to suppress 6-OHDA induced oxidative stress *in vitro* by various degrees in SH-SY5Y human cells pre-treated with GA and its derivatives. In addition to its protective effects in neurotoxicity models, GA counteracted the toxic effects of linden and carbon tetrachloride induced oxidative damage in the rat liver and kidneys (34,35). As shown in a study by Xu et al. (36), green tea polyphenols as potent antioxidants and free radical scavengers attenuated vascular cognitive impairment. 

A histological analysis and cell density measurements were used in the present study to explore the neuroprotective effects of GA on TMT toxicity in rats. Our findings indicated that administration of GA for two consecutive weeks prevented the sharp decline in cell density induced by TMT intoxication. Previous study showed that TMT induced neuronal death in the limbic system, with damage particularly to the hippocampus, and direct cytotoxic action on glial cells (37). Nilsberth et al. (38) reported extensive degeneration of pyramidal neurons caused by TMT intoxication in the CA3 region of the hippocampus plus neurodegeneration in the outer layer of the CA1 region and layer II of the entorhinal and piriform cortex. Results of immunoblot and immunocytochemical analyses indicated that TMT activated both caspase-3 and calpain. It also caused nuclear translocation of deoxyribonuclease II located within the cytoplasm in intact cells and could potentially damage neural cells (39). As previously mentioned, the decreased cell viability from TMT toxicity in rats occurred in parallel with decreased cell body size, increased DNA fragmentation, activation of caspase-9, and cleavage of the caspase substrate poly-ADP ribose polymerase (PARP) (25). In the current study, we determined the cell density and percentage of degenerated cells per unit area by morphological analyses of the hippocampal subdivisions as a marker of the neurotoxic effects of TMT. The results of this study demonstrated a significant increase in the percentage of degenerated cells in all hippocampal subdivisions of the TMT treated groups compared with intact controls. We observed that TMT intoxication decreased cell density in the CA1, CA2, CA3, and DG regions of the rats’ hippocampi as a marker of neurotoxic effects of TMT, which caused significant changes in mood and emotional states. 

## Conclusion

Our data demonstrated that treatment with GA could decrease depression and anxiety among TMT intoxicated rats. Mood stabilizing and neuroprotective effects of GA were found to be associated with the anti-oxidant and at least with amelioration of cell density loss in the hippocampus. However, our findings have not excluded other mechanisms involved in GA action. Further preclinical studies are necessary to elucidate possible functional mechanism(s) of this compound which include modulation of cholinergic and glutamatergic neurotransmission. Immunohistochemical analysis and evaluation of other parts of rat brain histomorphology in TMT toxic rats treated by GA are highly recommended as future research. Altogether, these findings may provide a pharmacological basis for GA to be used as an antioxidant metabolite for the treatment of neurodegenerative diseases such as hippocampal degeneration. 
